# Multisynaptic Inputs from the Medial Temporal Lobe to V4 in Macaques

**DOI:** 10.1371/journal.pone.0052115

**Published:** 2012-12-18

**Authors:** Taihei Ninomiya, Hiromasa Sawamura, Ken-ichi Inoue, Masahiko Takada

**Affiliations:** 1 Department of System Neuroscience, Tokyo Metropolitan Institute for Neuroscience, Fuchu, Tokyo, Japan; 2 Systems Neuroscience Section, Primate Research Institute, Kyoto University, Inuyama, Aichi, Japan; 3 Department of Ophthalmology, The University of Tokyo, Bunkyo-ku, Tokyo, Japan; University of Salamanca- Institute for Neuroscience of Castille and Leon and Medical School, Spain

## Abstract

Retrograde transsynaptic transport of rabies virus was employed to undertake the top-down projections from the medial temporal lobe (MTL) to visual area V4 of the occipitotemporal visual pathway in Japanese monkeys (*Macaca fuscata*). On day 3 after rabies injections into V4, neuronal labeling was observed prominently in the temporal lobe areas that have direct connections with V4, including area TF of the parahippocampal cortex. Furthermore, conspicuous neuron labeling appeared disynaptically in area TH of the parahippocampal cortex, and areas 35 and 36 of the perirhinal cortex. The labeled neurons were located predominantly in deep layers. On day 4 after the rabies injections, labeled neurons were found in the hippocampal formation, along with massive labeling in the parahippocampal and perirhinal cortices. In the hippocampal formation, the densest neuron labeling was seen in layer 5 of the entorhinal cortex, and a small but certain number of neurons were labeled in other regions, such as the subicular complex and CA1 and CA3 of the hippocampus proper. The present results indicate that V4 receives major input from the hippocampus proper via the entorhinal cortex, as well as “short-cut” pathways that bypass the entorhinal cortex. These multisynaptic pathways may define an anatomical basis for hippocampal-cortical interactions involving lower visual areas. The multisynaptic input from the MTL to V4 is likely to provide mnemonic information about object recognition that is accomplished through the occipitotemporal pathway.

## Introduction

Object recognition is a key function for the primate visual system. It has been revealed that recognizing visual objects is accomplished through the occipitotemporal pathway [1]–[4]. Visual area 4 (V4) that lies in an intermediate processing stage of the occipitotemporal pathway plays an important role in object recognition. V4 deals with basic components of objects such as form and color [5]–[9], and may encode the position of object parts relative to the focus of attention [10]. Furthermore, dysfunction of V4 leads to object recognition deficits [11]–[15]. While bottom-up sensory signals flow into higher visual areas of the temporal lobe for further processing, top-down signals primarily reach V4 and other lower visual areas to facilitate visual information processing. With regard to object recognition, mnemonic information should be utilized to identify what is present in the visual environment. In fact, learning and memory, which are possibly mediated by top-down signals, can affect the performance of visual tasks [16]. Neurophysiological and neuroimaging studies have also shown distinct effects of learning and memory on neuronal activities in many visual areas including V4 [17], [18].

Accumulated evidence to date indicates that the medial temporal lobe (MTL) is a critical structure for mnemonic functions, which include the capability of not only establishing long-term memory, but also retrieving necessary information from stored memory [19], [20]. Based on conventional anatomical studies, it has been revealed that, most of the visual input initially terminates in the parahippocampal and perirhinal cortices. Subsequently, the entorhinal cortex relays signals from the two cortical areas to the hippocampus. Major output from the hippocampus is in turn conveyed to the entorhinal cortex, then the parahippocampal and perirhinal cortices, and finally the visual areas where the inputs to the MTL originate [21]–[25]. Interactions between mnemonic and visual information processing are substantiated by the reciprocal linkage of the MTL and visual areas. While the top-down input from the MTL to V4 can provide information about learning and memory that obviously influence the function of V4 [17], [18], the MTL sends almost no direct input to V4 [26]. In the present study, multisynaptic inputs from the MTL to V4 were investigated by using the transneuronal tracing technique with rabies virus [27], [28].

## Materials and Methods

Five adult Japanese monkeys (*Macaca fuscata*) of either sex weighing 4−8 kg were used in this study. The experimental protocols were approved by the Animal Care and Use Committee of the Tokyo Metropolitan Institute for Neuroscience (Tokyo, Japan), and all experiments were conducted in line with the Guidelines for the Care and Use of Animals (Tokyo Metropolitan Institute for Neuroscience, 2000) (Permit Number: 08–1815). All efforts were made to minimize suffering: the monkeys were kept in individual primate cages in an air-conditioned room where food was always available. Their health conditions, including factors such as body weight and appetite, were checked daily. Supplementary water and fruit were provided daily. The details of the procedures for surgery, electrophysiological mapping, tracer injections, and histology were as described elsewhere [29].

### Viral and Tracer Injections

The challenge-virus-standerd (CVS-11) strain of rabies virus or conventional retrograde tracer wheat germ agglutinin-conjugated horseradish peroxidase (WGA-HRP) was injected into MT or V4 based on electrophysiological mappings. The virus was derived from the Centers for Disease Control and Prevention (Atlanta, GA, USA) and was donated by Dr. Satoshi Inoue (National Institute of Infectious Diseases, Tokyo, Japan). This strain was the same as that introduced by Ugolini [27] and Kelly and Strick [28] to demonstrate specific retrograde transsynaptic transport of the virus. The rate of retrograde transport for the viral batch used in this study was calibrated in our previous study [30]. By evaluating transneuronal labeling in the cortico-basal and cerebro-cerebellar loop circuits, we concluded that it takes about two days for first-order (monosynaptically connected) neuron labeling and one additional day per one synapse for subsequent transneuronal labeling. Moreover, the laminar distribution of neuronal labeling in V1 was analyzed in our recent study to confirm consistency with other studies on the primate visual system by means of rabies virus [29], [31], [32]. The titer of a viral suspension was 1.4×10^8^ focus-forming units/ml. The viral suspension was injected by pressure through a 10-µl modified Hamilton microsyringe that allows us to monitor neuronal activities immediately before the injection [33]. Three penetrations were typically made into V4 at least 1 mm apart from each other. For each penetration, 0.5–0.75 µl for rabies virus or 0.05–0.1 µl for WGA-HRP was deposited at two different depths (1–1.5 mm apart). Loci of the injection sites were arranged to cover multiple functional subregions within V4 at a given eccentricity (see Felleman et al. 1997 [34]; Xiao et al. 1999 [35]).

Rabies virus was injected into V4 of four Japanese monkeys. The monkeys were allowed to survive for 3 days (monkeys V4-3a and V4-3b) or 4 days (monkeys V4-4a and V4-4b). The details of the injection sites were as described elsewhere [29]. Only a brief account is provided here. Receptive fields (RFs) around the injection sites for monkeys V4-3a and V4-3b were in the lower visual quadrant at an eccentricity of approximately 5–15° and 15–40°, respectively. For monkeys V4-4a and V4-4b, RFs around the injection sites were in the lower visual quadrant at an eccentricity of approximately 10–40° and 5–10°, respectively. The distribution of neuronal labeling in the lateral geniculate nucleus and the primary visual cortex corresponded well with the extent of the injection sites, indicating that the rabies injections were made properly into V4. For the WGA-HRP injection case (monkey V4), RFs were located in the lower visual quadrant at an eccentricity of approximately 7–15°.

### Histological Procedures and Data Analysis

Three to four days after the rabies injections or four days after the WGA-HRP injections, the monkeys were deeply anesthetized with an overdose (50 mg/kg b.wt., i.v.) of sodium pentobarbital for perfusion-fixation. The monkeys were transcardially perfused with phosphate-buffered saline (PBS; 0.1 M, pH 7.4), followed by fixatives. The fixative for the monkeys with the rabies injections was a mixture of 10% formalin and 15% saturated picric acid in phosphate buffer (PB; 0.1 M, pH 7.4) and the one for the monkey with WGA-HRP injections was 8% formalin in 0.1 M PB. The brains were removed from the skull, postfixed in the same fresh fixative overnight, and saturated with 30% sucrose. Coronal sections were cut serially at 60 µm thickness on a freezing microtome. In the case of rabies injections, a series of every sixth section was immunohistochemically stained for rabies virus with the standard avidin-biotin peroxidase complex method as described elsewhere [29], [30]. These sections were counterstained with Cresyl violet or Neutral red. In the case of WGA-HRP injections, a series of every sixth section was reacted according to a tetramethylbenzidine-ammonium molybdate protocol [36]. A series of adjacent sections were Nissl-stained. Positions of labeled neurons were plotted with Neurolucida software (MicroBrightField, Williston, VT, USA). In both of the rabies and WGA-HRP injection cases, a series of sections was myelin-stained with a modified Gallyas method [37] to visualize laminar or areal borders.

The nomenclature of the MTL was adopted according to the parcellation of Amaral et al., [38], [39] and Suzuki and Amaral [40]. The MTL consists of the hippocampal formation, perirhinal cortex, and parahippocampal cortex. The hippocampal formation is composed of the dentate gyrus, the hippocampus proper (i.e., CA1 and CA3), the subicular complex (which can be further divided into the subiculum, presubiculum, and parasubiculum), and the entorhinal cortex. According to Rosene and Van Hoesen (1987) [41], the hippocampal formation was divided into the genu, uncus, anterior part, middle part, and posterior part through the longitudinal level. The perirhinal cortex is classified primarily into areas 35 and 36. To specify the temporal pole that has no direct connection with V4 [26], we further use “area TG” of von Bonin and Bailey [42]. The parahippocampal cortex is divided into areas TF and TH.

## Results

The direct (monosynaptic) projection from the temporal lobe to V4 was first evaluated in the WGA-HRP injection case (monkey V4; data not shown). Neuronal labeling was found in area TEO and the posterior and medial portions of area TE (TEp and TEm). In the MTL, retrogradely labeled neurons were confined to the posterior TF of the parahippocampal cortex, so-called visually responsive TF (VTF) [26], [43]. Neuronal labeling was sparse and was located mainly in deep layers. We did not observe labeled neurons in any other regions of the MTL, which was largely consistent with the study of Ungerleider et al. [26]. It should be noted here, however, that Ungerleider et al. [26] reported that they found a weak projection from the anterior portion of area TE (TEa) and TH to V4 in a few cases. The pattern of multisynaptic labeling in the MTL was then compared with that of monosynaptic labeling with WGA-HRP.

**Figure 1:**
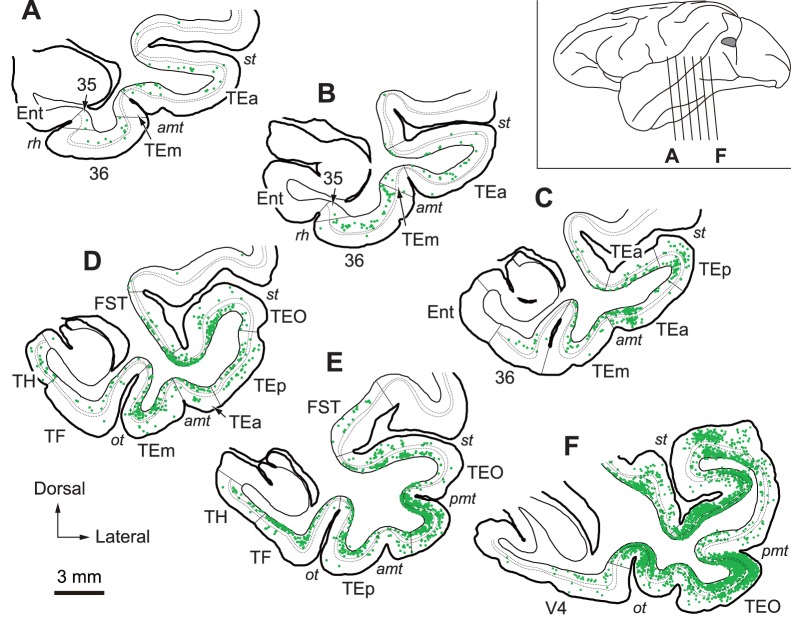
Distribution of retrograde labeling in the MTL 3 days after rabies injections into V4. Six representative coronal sections through the MTL in monkey V4-3a are arranged anteroposteriorly (A–F). The approximate anteroposterior levels of the sections are indicated in the lateral view of the brain (inset). The gray region represents the approximate whole extent of multiple injection sites in monkey V4-3a. Each green dot in the sections (A–F) corresponds to one labeled neuron. Dotted lines denote the borders of layer 4 in each area. amt, anterior middle temporal sulcus; Ent, entorhinal cortex; FST, fundus of the superior temporal sulcus area; ot, occipitotemporal sulcus; pmt, posterior middle temporal sulcus; rh, rhinal sulcus; st, superior temporal sulcus; TEa, anterior portion of area TE; TEm, medial portion of area TE; TEO, area TEO; TEp, posterior portion of area TE; TF, area TF; TH, area TH; 35, area 35; 36, area36.

On day 3 after the rabies injections into V4, neuronal labeling occurred in many temporal lobe areas, i.e., all areas that have been reported to possess monosynaptic connections with V4, including area TEO, all subdivisions of area TE (TEp, TEm, and TEa), and the parahippocampal cortex (areas TF and TH) [26], [44]. Labeled neurons in the parahippocampal cortex were seen mainly in its posterior portion, and neuronal labeling in area TF was observed relatively stronger in the medial portion than in the lateral portion (Fig. 1E; monkey V4-3a). Furthermore, substantial neuron labeling appeared in area 36 of the perirhinal cortex, and, additionally, a small number of labeled neurons were seen in area 35 of the perirhinal cortex (Fig. 1). In view of the fact that areas 35 and 36 do not have direct connections with V4 [26], such rabies labeling of perirhinal cortical neurons corresponds to disynaptic neuron labeling. The labeled neurons in these areas were located predominantly in deep layers of the caudal part. In area 36, neuronal labeling was very weakly biased toward its lateral portion, especially at the caudal level. Disynaptic inputs might be expected from the entorhinal cortex, subicular complex, or CA1 to V4, because the parahippocampal cortex and area TE have been reported to connect with these areas [22], [45], [46]. However, essentially no rabies-labeled neurons appeared in the hippocampal formation. It should be noted here that neuronal labeling was observed extensively over the amygdaloid complex. The labeled neurons were located most densely in the basolateral nucleus that is known to project directly to V4 [47]. In addition, rabies-labeled neurons were distributed in the most part of the amygdaloid complex, including the accessory basal nucleus, the cortical nuclei, the cortical-amygdaloid transition area, and the basomedial nucleus, in which projections from the subicular complex and CA1 terminate [48].

**Figure 2:**
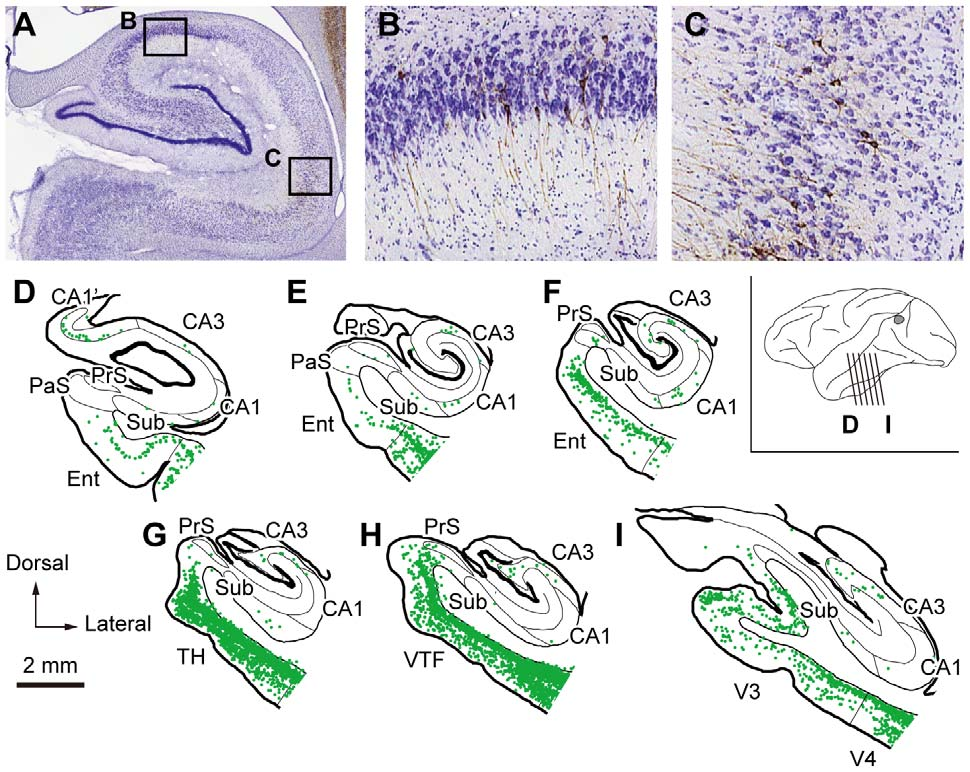
Distribution of retrograde labeling in the MTL 4 days after rabies injections into V4. A , Photomicrograph of rabies-labeled neurons in the hippocampal formation, counterstained with Cresyl violet. **B, C**, Higher-magnification images of the labeled neurons (brown) in CA3 (B) and CA1 (C), taken from the rectangular areas in A. **D–I**, Six representative coronal sections through the hippocampal formation in monkey V4-4b are arranged anteroposteriorly. PaS, parasubiculum; PrS, presubiculum; Sub, subiculum; V3, visual area 3; VTF, visually responsive TF. Other conventions and abbreviations are as in Figure 1.

To identify possible multisynaptic projections from the hippocampal formation to V4, two monkeys were allowed to survive for 4 days after the rabies injections into V4. On day 4, a tremendous number of labeled neurons were observed in the temporal lobe. This suggested that further transsynaptic transport of rabies virus occurred during one additional survival day. In the MTL, neuronal labeling was evident in superficial and deep layers of the parahippocampal (areas TF and TH) and perirhinal (areas 35 and 36) cortices. Strong neuron labeling was also seen in area TG (data not shown). Moreover, we found substantial neuron labeling in the hippocampal formation (Fig. 2; monkey V4-4b). The entorhinal cortex contained a large number of labeled neurons, mostly in layer 5 through the rostrocaudal axis. Neuronal labeling was the heaviest in the posterior part, which contained the caudal limiting field and the posterior part of the caudal field of the entorhinal cortex as designated in the study of Amaral et al. (1987) [39]. In addition, a small but certain number of neurons were labeled in the pyramidal cell layer of CA1 and CA3 and all subdivisions of the subicular complex (i.e., the subiculum, presubiculum, and parasubiculum). Neuronal labeling in the hippocampus proper (CA1 and CA3) and the subicular complex was seen mainly in the middle and posterior parts. In the coronal plane, labeled neurons were located in the proximal CA1 near the subiculum. Neuronal labeling was also observed in the dorsal part of the rostral CA1, corresponding to CA1’ as designated in the study of Saunders et al. (1988) (Fig. 2D) [48]. On the other hand, the dentate gyrus was virtually devoid of neuronal labeling. Although rabies virus was injected into two V4 regions representing different eccentricities in the two animals with survival period of 3 days (monkey V4-3a, 5°–15°; monkey V4-3b, 15°–40°) or 4 days (monkey V4-4a, 10°–40°; monkey V4-4b, 5°–10°), no clear differences were observed in the distribution of neuronal labeling in the MTL.

**Figure 3:**
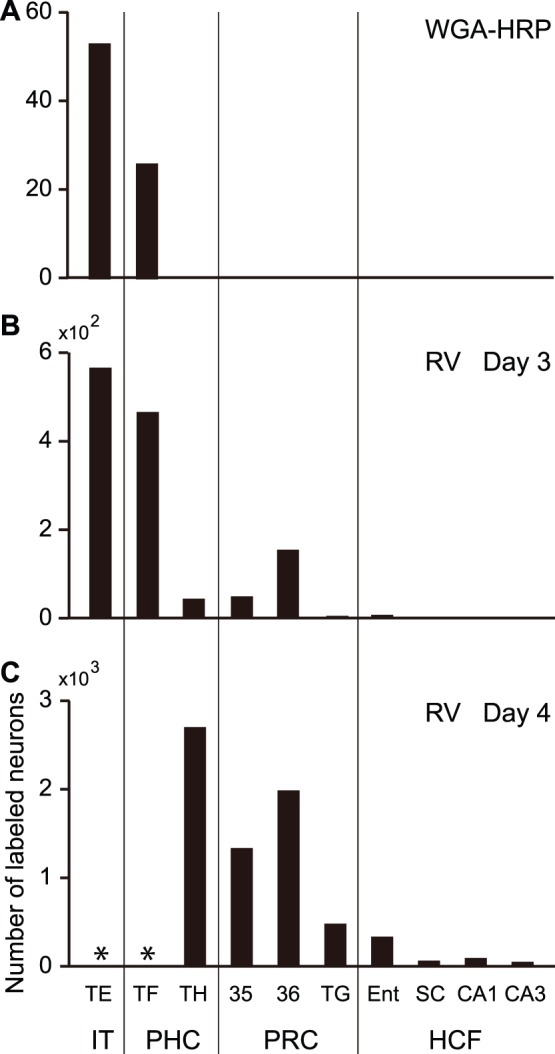
Histograms showing the differences in retrograde labeling distributions in the inferotemporal cortex (area TE) and the MTL. A, Case of WGA-HRP injections into V4. B, Case of rabies injections into V4 at the 3-day postinjection period. C, Case of rabies injections into V4 at the 4-day postinjection period. Cell counts were performed in every 12th section (60 µm thick; 720 µm apart). Data from the WGA-HRP injection case were obtained in monkey V4, while those taken at the 3- or 4-day postinjection period represent the average of the labeled neuron numbers in monkeys V4-3a and V4-3b or monkeys V4-4a and V4-4b, respectively. Asterisks indicate the areas in which cell counts could not be done, as a tremendous number of rabies-labeled neurons appeared diffusely. The subicular complex (SC) consists of the subiculum, presubiculum, and parasubiculum. Each of these regions contained are 34, 15, and 12, respectively). HCF, hippocampal formation; IT, inferotemporal cortex; PHC, parahippocampal cortex; PRC, perirhinal cortex; TG, area TG. Other abbreviations are as in Figures 1 and 2.

Figure 3 shows the distribution patterns of labeled neurons in the MTL after the WGA-HRP and rabies injections into V4: (1) Neurons projecting directly to V4 existed only in area TF of the parahippocampal cortex; (2) Three days after the rabies injections, transneuronal labeling was evident in area TH of the parahippocampal cortex and areas 35 and 36 of the perirhinal cortex, but not in the hippocampal formation; (3) At the 4-day postinjection period, rabies-labeled neurons appeared in the subicular complex, CA1, and CA3 of the hippocampal formation.

## Discussion

The present results provide evidence that the parafoveal to peripheral part of V4 receives ample information from the hippocampal formation as early as in a trisynaptic manner. On day 3 after the rabies injections into V4, labeled neurons appeared in areas TF and TH of the parahippocampal cortex and areas 35 and 36 of the perirhinal cortex. The other components of the MTL are virtually devoid of neuronal labeling. Lack of direct projections from the perirhinal cortex to V4 [26], as well as an internal control tested in our laboratory [30], indicates that the second-order neuron labeling occurs at the 3-day survival period. On day 4 after the rabies injections into V4, further neuron labeling emerged prominently in layer 5 of the entorhinal cortex, with fewer labeling in the subicular complex and CA1 presumably in a trisynaptic fashion (for detail, see Materials and Methods). However, it is difficult to argue with confidence that the third-order neuron labeling occurred in CA3 at the 4-day survival period. Neuronal labeling in CA3 must have occurred subsequently to neuronal labeling in other part(s) of the hippocampal formation, because CA3 does not send output outside the hippocampal formation. However, virtually no labeling was observed in the hippocampal formation on day 3 after the rabies injections. Two interpretations can be postulated for this contradiction. One is that rabies virus was transsynaptically transported faster than expected after the infection of CA1 neurons, as the virus might travel with a brief period through internal connections between CA1 and CA3. The other interpretation is that neurons in CA3 indeed project outside the hippocampal formation, and rabies virus traveled so-far-unappreciated route(s).

**Figure 4:**
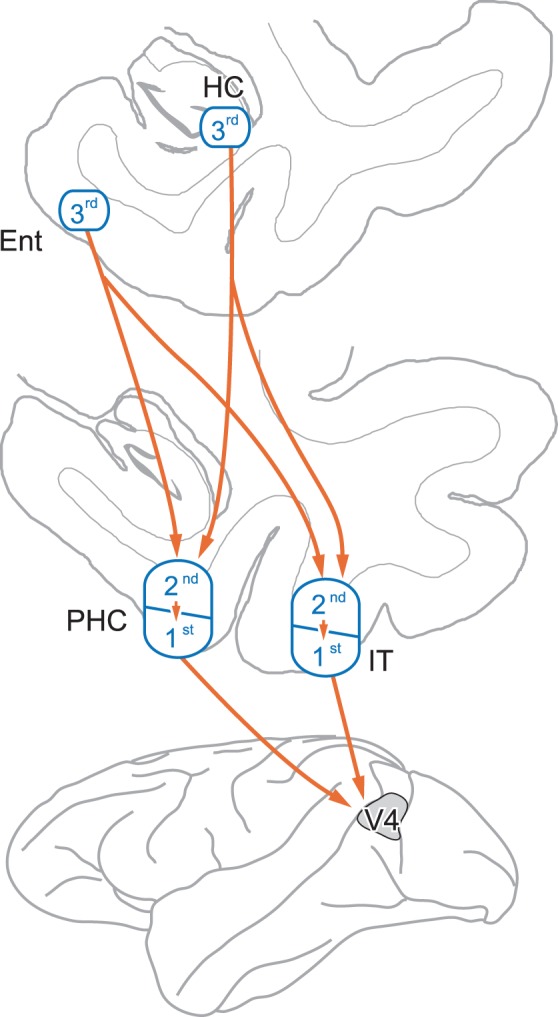
Summary diagram showing possible multisynaptic pathways from the MTL to V4. Our results indicate that the entorhinal cortex (Ent) provides trisynaptic input to V4 via either the parahippocampal cortex (PHC) or the inferotemporal cortex (IT), while the hippocampus (HC) does through “short-cut” pathways that bypass the Ent, as denoted by orange arrows. The gray area represents the approximate location whole extent of the rabies injection sites in 4 monkeys used for the present study. For clarity, only presumably major pathways are shown here (see Discussion for details). HC, hippocampus; 1st, first-order neurons; 2nd, second-order neurons; 3rd, third-order neurons.

As shown in Figure 4, we propose possible multisynaptic pathways from the MTL to V4. Dense neuron labeling in the entorhinal cortex suggests that the entorhinal cortex mainly provides information derived from the hippocampus to visual areas, as described in many previous studies [20], [22], [24], [25]. The trisynaptic input from the entorhinal cortex is likely conveyed via the parahippocampal, and/or inferotemporal cortical areas and their internal circuits. At the same timing, sparse neuron labeling was detected in the subicular complex and CA1 and CA3 of the hippocampus proper. Such neuronal labeling is probably ascribable to transsynaptic transport of rabies virus through “short-cut” pathways that bypass the entorhinal cortex. Similar to the trisynaptic input from the entorhinal cortex, the parahippocampal and/or inferotemporal cortical areas are likely involved in these trisynaptic bypass routes. Though less likely, the neuronal connections of the perirhinal cortex and between the parahippocampal and the inferotemporal cortex might participate in any of the trisynaptic pathways.

V4 connects prominently with area TE of the inferotemporal cortex and area TF of the parahippocampal cortex [26], both of which receive inputs directly from the hippocampal formation [45], [46]. Based on this fact, it is somewhat surprising that the second-order neuron labeling did not occur in the hippocampal formation. Since the rate of rabies transport is thought to depend on the strength of connectivity [27], there is a possibility that we may have missed labeled neurons in the hippocampal formation at the 3-day survival period. However, this seems unlikely because neuronal connections between V4 and TF and between TF and CA1 are fairly reliable [26], [43], [46]. Thus, the present results imply that further processing may take place within the inferotemporal and parahippocampal cortices to convey signals from the hippocampus to V4. Otherwise, the perirhinal cortex may participate in the trisynaptic pathways from the hippocampal formation to V4. There is another possibility that internal connections between the inferotemporal and the parahippocampal cortex may be involved in the trisynaptic pathways. Besides, the possible involvement of the amygdaloid complex should be taken into consideration. The basolateral nucleus, especially its dorsal part, projects to V4 [47], while other nuclei (accessory basal nucleus, cortical nuclei, cortical-amygdaloid transition area, and basomedial nucleus) receive inputs from CA1’ and the subicular complex [48]. Actually, we did find labeled neurons in these nuclei 3 days after the rabies injections into V4. Thus, rabies labeling in the hippocampal formation, especially CA1’, may be ascribable to multisynaptic pathways mediated by the amygdala. This is of particular interest, as functionally distinct inputs arising from the hippocampus may simultaneously be directed toward V4 through different pathways.

The multisynaptic input from the MTL possibly confers mnemonic information to V4. Such information might greatly help to interpret an incoming visual stimulus, which is useful when the stimulus is ambiguous. In fact, neuronal activity in V4 shows an enhancement for degraded familiar stimuli, thus indicating that top-down signals can play a more important role than bottom-up signals [17]. Furthermore, connectivity between the MTL and V4 might contribute not only to cognitive function in an awake condition, but also to the process of off-line memory consolidation. It has been reported in the rat that coordinated replay of awake experience during sleep occurs in the hippocampus and the primary visual cortex [49]. The coordination of neuronal activities presumably requires active communication between the hippocampus and the cortex. The multisynaptic pathways identified in the present study may define part of anatomical basis for such hippocampal-cortical interactions involving lower visual areas. Taken together, the MTL likely provides top-down signals that are related to mnemonic functions to V4 multisynaptically.
